# Influence of different treatment methods of Left Colic Artery on postoperative rehabilitation of patients undergoing Laparoscopic Radical Resection of Rectal Cancer

**DOI:** 10.12669/pjms.39.1.6366

**Published:** 2023

**Authors:** Chuang Ding, Lingyong Kong, Ming Zhang, Yan Chen

**Affiliations:** 1 Chuang Ding, Department of Gastrointestinal Surgery, The Affiliated Suqian Hospital of Xuzhou Medical University, Suqian 223800, Jiangsu, China; 2 Lingyong Kong, Department of Gastrointestinal Surgery, The Affiliated Suqian Hospital of Xuzhou Medical University, Suqian 223800, Jiangsu, China; 3 Ming Zhang, Department of Gastrointestinal Surgery, The Affiliated Suqian Hospital of Xuzhou Medical University, Suqian 223800, Jiangsu, China; 4 Yan Chen, Department of Gastrointestinal Surgery, The Affiliated Suqian Hospital of Xuzhou Medical University, Suqian 223800, Jiangsu, China

**Keywords:** Left colic artery, Laparoscopic radical resection of rectal cancer, Enhanced recovery after surgery

## Abstract

**Objective:**

To investigate the effect of different treatment methods of the left colic artery (LCA) on postoperative rehabilitation of patients undergoing laparoscopic radical resection of rectal cancer.

**Methods:**

Retrospective analysis was performed on 70 patients undergoing laparoscopic radical resection of rectal cancer who were admitted to The Affiliated Suqian Hospital of Xuzhou Medical University from January, 2020 to December, 2022 were selected and divided into two groups according to different treatment methods of LCA. The preservation group (LCA group) (n=34 cases) and the non-preservation group (NLCA group) (n=36 cases). Both groups were treated with laparoscopic radical resection of rectal cancer. IMA was preserved in the LCA group, but not in the NLCA group. The efficacy indicators, surgical treatment and rehabilitation-related indicators, gastrointestinal hormone indicators (motilin (MTL), gastrin (GAS)), and postoperative complications risk were compared between the two groups before and after surgery.

**Results:**

No statistically significant difference was observed between the two groups in terms of efficacy indicators (total number of lymph nodes dissected and number of lymph nodes at the root of the IMA), operation time, intraoperative blood loss, and postoperative drainage tube placement time (p>0.05). However, postoperative anal flatus and hospital stay in the LCA group were considerably shorter than those in the NLCA group (p<0.05). Postoperatively, the levels of MTL and GAS in the two groups were significantly decreased, and the LCA group decreased slightly compared with the NLCA group (p<0.05). Moreover, the incidence of complications in the LCA group (5.88%) was significantly lower than that in the NLCA group (27.78%) (p<0.05).

**Conclusion:**

Preservation of LCA and no-preservation of LCA in laparoscopic radical resection of rectal cancer are comparable in terms of therapeutic effect, and the surgery with preservation of LCA is worthy of clinical promotion due to its various benefits such as less impact on gastrointestinal hormone indicators, lower risk of complications, and faster postoperative recovery.

## INTRODUCTION

Rectal cancer is a malignant tumor with a high incidence and high mortality in China, and its diagnosis and treatment research is highly valued in clinical practice. Currently, a comprehensive treatment strategy based on the concept of early diagnosis and surgery is preferred for the treatment of rectal cancer. Laparoscopic radical resection of rectal cancer is the main clinical treatment at this stage, with definite curative effect and less trauma.^1^-^3^ Radical surgery can be divided into low and high ligation according to the different treatment methods of the inferior mesenteric artery (IMA) and its branches, among which low ligation is based on IMA ligation and left colonic artery (LCA) preservation, while high ligation is based on IMA root ligation and LCA removal.^4^ Considering the respective advantages of preservation of LCA and no-preservation of LCA, for example, no-preservation of LCA allows a wider scope of lymph node dissection, while preservation of LCA has been proved by many studies to have a variety of therapeutic advantages, which is conducive to ameliorating the blood supply of proximal anastomosis, and achieving a scope of lymph node dissection similar to that no-preservation of LCA.^5^-^6^ Nevertheless, controversy still exists in the current clinical practice about whether to preserve LCA in radical surgery. Whether to preserve LCA has not been explicitly mentioned in either the National Comprehensive Cancer Network or the Japanese Society for Cancer of the Colon and Rectum Guidelines.^7^ In addition, more high-quality medical evidence is needed to determine whether the preservation of LCA will affect patient survival and reduce the risk of complications. Based on this, an analysis was conducted on the influence of different treatment methods of LCA on postoperative rehabilitation of patients undergoing laparoscopic radical resection of rectal cancer, aiming to provide patients with effective and safe treatment strategies.

## METHODS

### General Information:

Retrospective analysis was performed on 70 patients undergoing laparoscopic radical resection of rectal cancer who were admitted to The Affiliated Suqian Hospital of Xuzhou Medical University from January, 2020 to December, 2022 were selected. They were divided into two groups according to different treatment methods of LCA: the preservation group (LCA group) (n=34 cases) and the non-preservation group (NLCA group) (n=36 cases). In the LCA group, there were 21 males and 13 females, aged 34-79 years (58.42±11.28), with a tumor diameter of 2-9cm (5.41±1.35), and tumor stage of Stage-I in nine cases, Stage-II in 15 cases, and Stage-III in 10 cases. In the NLCA group, there were 22 males and 14 females, aged 36-77 years (58.13±11.47), with a tumor diameter of 2-8 cm (5.32±1.44), and tumor stage of Stage-I in 10 cases, Stage-II in 17 cases, and Stage-III in nine cases. No statistically significant difference was observed in the general data (gender, age, tumor diameter and stage) between the two groups (p>0.05).The study was approved by the Institutional Ethics Committee of The Affiliated Suqian Hospital of Xuzhou Medical University (No.: 2022-0322; Date: April 15, 2022), and written informed consent was obtained from all participants.

### Inclusion Criteria:

- Patients with stage I, II, and III colorectal cancer who met the surgical indication criteria in the “Guideline for Operative Procedure of Laparoscopic Radical Resection of Colorectal Cancer”^8^.
- Patients with no history of abdominal surgery;
- Patients who signed informed consent.

### Exclusion Criteria:

- Patients with contraindications to laparoscopic radical resection of rectal cancer: inability to establish pneumoperitoneum, pregnancy, poor systemic status and unable to correct, or unable to tolerate surgery;
- Patients with extensive tumor infiltration into surrounding tissues;
- Patients with severe heart, lung, liver and kidney diseases.

Both groups were given laparoscopic radical resection of rectal cancer, with the same preoperative preparation, including intestinal preparation, imaging examination, etc. Patients were guided to a modified lithotomy position with head-low and foot-high position maintained at 15º, and a conventional pneumoperitoneum was established using the four-hole method (12mm observation hole (location: 1cm above the umbilicus), 12mm main operating hole (location: McBurby’s point of the right lower abdomen), and two 5cm operation holes (location: left and right center of abdominal clavicle at umbilical level)). After a thorough examination of the pelvic and abdominal cavity, an incision was made on the medial side of the right internal iliac artery, which was dissociated cephalad from the sigmoid mesocolon serosa to the root of the IMA. The IMA was preserved in the LCA group as follows: Firstly, the lymph nodes at the root of the IMA were identified, and the vascular surface tissues were stripped along the IMA to fully reveal the LCA root. Then, vascular surface tissues were stripped along the LCA until the intersection of inferior mesenteric veins, and complete dissection was performed on the lymph nodes between the right side and root of the IMA, the left side of the inferior mesenteric vein and the LCA (including the third-order lymph nodes). Finally, a distal ligation was performed 1cm below the LCA crossover site extending from the IMA. After the resection, a drainage tube and an anal tube were indwelled at the anastomosis, and the abdominal cavity was closed layer by layer.

IMA was not preserved in the NLCA group.The dissociation method of IMA and the operation prior to dissociation were the same as those in the LCA preservation group. After dissociating to the root of IMA, ligation was performed at the 1cm site extending from the abdominal aorta, which was dissociated to the left for dissection of the inferior mesenteric vein, and the surrounding lymph nodes (including the third-node lymph nodes) were dissected. A 5-8cm incision was made in the middle of the subumbilical abdomen, and the dissociated bowel and the corresponding mesentery were taken out. Then, dissociation ligation was performed, with the arterial arch of the colon preserved. The postoperative operation was the same as that of the LCA preservation group.

### Observation Indicators:

Comparison of efficacy indicators between the two groups: the total number of dissected lymph nodes and the number of lymph nodes at the root of the IMA were recorded.

Comparison of surgical treatment and rehabilitation-related indicators between the two groups: the operation time, intraoperative blood loss, postoperative anal exhaust, drainage tube placement time and hospital stay were recorded.

Comparison of gastrointestinal hormone indicators between the two groups: these indicators were evaluated before and after surgery. Four mililiter venous blood was collected from patients in a fasting state in the morning, and the levels of motilin (MTL) and gastrin (GAS) were detected by radioimmunoassay (kit from Hangzhou Haoxin Biotechnology Co., Ltd).

Comparison of the risk of postoperative complications between the two groups: the risks of postoperative complications such as urinary retention, intestinal obstruction, anastomotic leakage and bleeding were recorded.

### Statistical Methods:

All data were analyzed with SPSS23.0 software. Independent sample t test was used for the comparison of measurement data (data consistent with normal distribution) between the two groups, paired sample t test was used for preoperative and postoperative comparison, represented by (*χ̄*±*S*), and χ^2^ test was used for counting data, represented by rate (%). P*<*0.05 indicates a statistically significant difference.

## RESULTS

**Fig.1:**
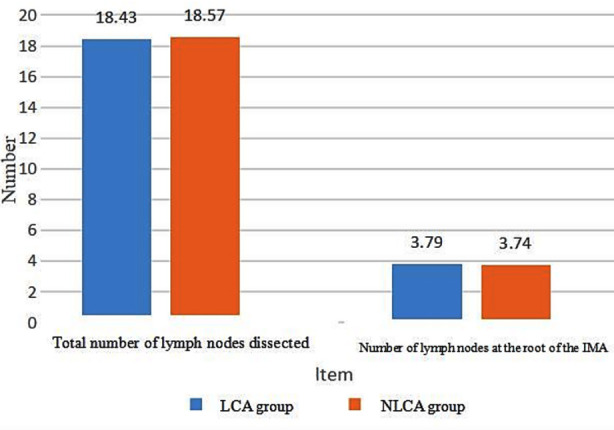
Comparison of efficacy indicators between the two groups.

No statistically significant difference was observed between the two groups in terms of efficacy indicators (total number of lymph nodes dissected and number of lymph nodes at the root of the IMA).Table-I, Fig.1.

**Table-I:** Comparison of the efficacy indicators between the two groups (*χ̅*±*S*).

Group	Number of cases	Total number of lymph nodes dissected	Number of lymph nodes at the root of the IMA
LCA group	34	18.43±2.52	3.79±0.82
NLCA group	36	18.57±2.41	3.74±0.91
t	-	0.238	0.241
P	-	0.813	0.810

No statistically significant difference was observed between the two groups in terms of the operation time, intraoperative blood loss, and postoperative drainage tube placement time (*p>*0.05). However, postoperative anal flatus and hospital stay in the LCA group were considerably shorter than those in the NLCA group (*p<*0.05). Table-II.

**Table-II:** Comparison of surgical treatment and rehabilitation-related indicators between the two groups (*χ̅*±*S*).

Group	Number of cases	Operation time (min)	Intraoperative blood loss (ml)	Anal exhaust time (h)	Drainage tube placement time (d)	Hospital stay (d)
LCA group	34	149.69±35.38	53.67±4.32	21.34±2.35	7.24±1.39	9.03±1.58
NLCA group	36	134.57±32.18	54.01±4.06	24.58±2.39	7.27±1.31	10.57±1.84
t	-	1.872	0.340	5.715	0.093	3.747
P	-	0.066	0.735	0.001	0.926	0.001

**Fig.2:**
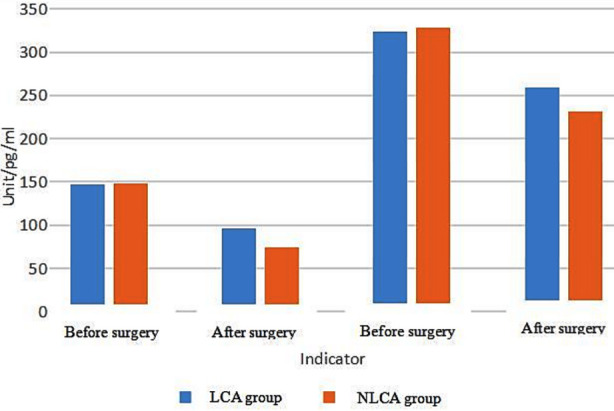
Comparison of gastrointestinal hormone indicators between the two groups.

No statistically significant difference was observed in MTL and GAS between the two groups before surgery (*p>*0.05); Postoperatively, the levels of MTL and GAS in the two groups were significantly decreased, and the LCA group decreased slightly compared with the NLCA group (*p<*0.05). Table-III, Fig.2. The incidence of complications in the LCA group (5.88%) was significantly lower than that in the NLCA group (27.78%) (*p<*0.05). Table-IV.

**Table-III:** Comparison of gastrointestinal hormone indicators between the two groups (*χ̅*±*S*).

Group	Number of cases	MTL (pg/ml)		GAS (pg/ml)	
	
		Before surgery	After surgery	Before surgery	After surgery
LCA group	34	147.53±25.47	96.49±11.25*	324.17±39.48	259.64±23.27*
NLCA group	36	148.03±23.25	74.32±10.34*	328.14±37.11	231.37±21.08*
t	-	0.086	8.591	0.434	5.332
P	-	0.932	0.001	0.666	0.001

***Note:***

* *P* indicates p<0.05 compared with preoperative.

**Table-IV:** Comparison of the risk of postoperative complications between the two groups [n, (%)].

Group	Number of cases	Urinary retention	Intestinal obstruction	Anastomotic leakage	Anastomotic bleeding	Incidence
LCA group	34	0 (0.00)	1 (2.94)	1 (2.94)	0 (0.00)	2 (5.88)
NLCA group	36	1 (2.78)	1 (2.78)	5 (13.89)	3 (8.33)	10 (27.78)
χ^2^	-	-	-	-	-	4.461**
P	-	-	-	-	-	0.035

***Note:***

** means continuous χ^2^ correction.

## DISCUSSION

Laparoscopic radical resection of rectal cancer, as a commonly used minimally invasive surgery for rectal cancer, is performed under laparoscopic direct vision, with efficacy equivalent to open surgery in terms of resection of the tumor and surrounding tissue, and control of disease progression. It boasts various benefits such as protection of perioperative tissues and nerves, less trauma, reduced inflammatory response, reduced risk of traumatic surgery on immune damage and impact on gastrointestinal function, and higher safety.^9^,^10^ However, there is a controversy in clinical practice regarding different treatment methods (preservation or non-preservation) of the left colic artery: whether the risk of postoperative complications and functional impairment can be reduced under the premise of ensuring the outcome of oncological treatment.^11^

Non-preservation of LCA boasts therapeutic advantages in that lymph node dissection can be performed at all sites of rectal cancer to the IMA root region, minimizing the risk of residual positive lymph nodes. However, Non-preservation of LCA may inadvertently injure the branches and nerves of the IMA due to the large anatomical variation of the LCA, thereby affecting the completeness of lymph node dissection. In addition, cutting the LCA may result in insufficient blood supply to the colon, an increased risk of complications, and a forced extension of resection and tension at the anastomotic site. In contrast, preservation of LCA is a treatment method proposed to be different from non-preservation of LCA in clinical practice, which can avoid the adverse effects after LCA removal. However, its therapeutic effect and safety remain to be demonstrated in more trials.^12^ It was shown in this study that there were no statistically significant differences observed between the two groups in terms of efficacy indicators (total number of lymph nodes dissected and number of lymph nodes at the root of the IMA), operation time, intraoperative blood loss, and postoperative drainage tube placement time (*p>*0.05). However, postoperative anal flatus and hospital stay in the LCA group were considerably shorter than those in the NLCA group (p<0.05). With the continuous improvement of technology, laparoscopic surgery has continuously reduced limitations in the surgical field and operating space, which is more conducive to improving the therapeutic effect of preservation of LCA and avoiding excessive colon resection due to non-tumor factors. Therefore, the surgical effects of the LCA group and the NLCA group were similar. In the NLCA group, the long-term insufficiency of blood supply to the proximal colon weakened gastrointestinal peristalsis and affected postoperative recovery time.^13^ While in the LCA group, rectal blood perfusion was more adequate, which reduced the risk of gastrointestinal spasm or slow peristalsis caused by intestinal ischemia, and accelerated recovery of anal exhaust and hospital stay.^14^ It was shown in the study of Sun K et al.^15^ that the preservation of LCA can shorten the time of initial ventilation and accelerate the recovery, which is consistent with the results of this study. Postoperatively, the levels of MTL and GAS in the two groups were significantly decreased, and the LCA group decreased slightly compared with the NLCA group (p<0.05). The reason can be attributed to the fact that radical surgery without preserving LCA will damage the autonomic nerve at the root of the IMA, showing urogenital dysfunction and affecting gastrointestinal hormone indicators. In contrast, surgery with LCA preservation can better protect gastrointestinal function, avoid the risk area of pelvic autonomic nerve injury, and promote gastrointestinal function recovery. The incidence of complications in the LCA group (5.88%) was significantly lower than that in the NLCA group (27.78%) (*p<*0.05). The reason can be attributed to the fact that the preservation of LCA is more conducive to the anastomotic blood supply and reduces the risk of anastomotic leakage and bleeding. However, there was little difference between the two groups in the risk of complications such as urinary retention and intestinal obstruction. No statistically significant difference was observed in the comparison of lymph node dissection and operation time between the two groups.

### Limitations of the study:

However, compared with surgery without LCA preservation, surgery with LCA preservation had a lower risk of anastomotic fistula and other complications, and was more consistent with the concept of accelerated rehabilitation surgery and shorter hospital stay. In follow-up studies, more samples need to be included to clarify the safety and efficacy indicators of the surgery with LCA preservation. In addition, patients with the poor vascular condition, advanced age, and late tumor stage are not recommended to perform surgery with LCA preservation. In the future, more studies are needed to analyze and expand the application scope of surgery with LCA preservation.

## CONCLUSIONS

Preservation of LCA and non-preservation of LCA in laparoscopic radical resection of rectal cancer are comparable in terms of therapeutic effect, and the surgery with preservation of LCA is worthy of clinical promotion due to its various benefits such as less impact on gastrointestinal hormone indicators, lower risk of complications, and faster postoperative recovery.

### Authors’ Contributions:

**CD & MZ:** Designed this study and prepared this manuscript, are responsible and accountable for the accuracy and integrity of the work.

**LK:** collected and analyzed clinical data.

**YC:** Data analysis**,** Significantly revised this manuscript.
